# NADPH Oxidase Subunit 4-Mediated Reactive Oxygen Species Contribute to Cycling Hypoxia-Promoted Tumor Progression in Glioblastoma Multiforme

**DOI:** 10.1371/journal.pone.0023945

**Published:** 2011-09-15

**Authors:** Chia-Hung Hsieh, Woei-Cherng Shyu, Chien-Yi Chiang, Jung-Wen Kuo, Wu-Chung Shen, Ren-Shyan Liu

**Affiliations:** 1 Graduate Institute of Basic Medical Science, China Medical University and Hospital, Taichung, Taiwan; 2 Department of Neurology, Center for Neuropsychiatry, and Graduate Institute of Immunology, China Medical University and Hospital, Taichung, Taiwan; 3 Graduate Institute of Clinical Medical Science, China Medical University, Taichung, Taiwan; 4 Department of Nuclear Medicine, School of Medicine, School of Biomedical Science and Engineering, National Yang Ming University, Taipei, Taiwan; 5 Department of Radiology, China Medical University and Hospital, Taichung, Taiwan; 6 National PET/Cyclotron Center, Department of Nuclear Medicine, Taipei Veterans General Hospital, Taipei, Taiwan; Faculdade de Medicina, Universidade de São Paulo, Brazil

## Abstract

**Background:**

Cycling and chronic tumor hypoxia are involved in tumor development and growth. However, the impact of cycling hypoxia and its molecular mechanism on glioblastoma multiforme (GBM) progression remain unclear.

**Methodology:**

Glioblastoma cell lines, GBM8401 and U87, and their xenografts were exposed to cycling hypoxic stress *in vitro* and *in vivo*. Reactive oxygen species (ROS) production in glioblastoma cells and xenografts was assayed by *in vitro* ROS analysis and *in vivo* molecular imaging studies. NADPH oxidase subunit 4 (Nox4) RNAi-knockdown technology was utilized to study the role of Nox4 in cycling hypoxia-mediated ROS production and tumor progression. Furthermore, glioblastoma cells were stably transfected with a retroviral vector bearing a dual reporter gene cassette that allowed for dynamic monitoring of HIF-1 signal transduction and tumor cell growth *in vitro* and *in vivo*, using optical and nuclear imaging. Tempol, an antioxidant compound, was used to investigate the impact of ROS on cycling hypoxia-mediated HIF-1 activation and tumor progression.

**Principal Findings:**

Glioblastoma cells and xenografts were compared under cycling hypoxic and normoxic conditions; upregulation of NOX4 expression and ROS levels were observed under cycling hypoxia in glioblastoma cells and xenografts, concomitant with increased tumor cell growth in *vitro* and *in vivo*. However, knockdown of Nox4 inhibited these effects. Moreover, *in vivo* molecular imaging studies demonstrated that Tempol is a good antioxidant compound for inhibiting cycling hypoxia-mediated ROS production, HIF-1 activation, and tumor growth. Immunofluorescence imaging and flow cytometric analysis for NOX4, HIF-1 activation, and Hoechst 3342 in glioblastoma also revealed high localized NOX4 expression predominantly in potentially cycling hypoxic areas with HIF-1 activation and blood perfusion within the endogenous solid tumor microenvironment.

**Conclusions:**

Cycling hypoxia-induced ROS via Nox4 is a critical aspect of cancer biology to consider for therapeutic targeting of cycling hypoxia-promoted HIF-1 activation and tumor progression in GBM.

## Introduction

Tumor hypoxia is a crucial microenvironmental condition that promotes tumor progression and resistance to chemo- or radiotherapy [1], [2], [3]. It has been classified into 2 types. Acute hypoxia is associated with inadequate blood flow while chronic hypoxia is the consequence of increased oxygen diffusion distance due to tumor expansion. Temporal instability in oxygen transport has classically been termed “intermittent” or “acute” hypoxia [4]. Recent review articles have summarized the overall features of the oxygenation state within tumors and have used the term “cycling hypoxia” to describe the cyclical features of intermittent or acute hypoxia in tumor hypoxia [5]. It has long been thought that chronic hypoxia, rather than acute hypoxia, plays the main role in promotion of cancer progression and in the efficacy of radiation therapy or chemotherapy [1], because the major phenotypic shift associated with chronic hypoxia involves tumor cell resistance to chemotherapy or radiotherapy, in addition to more invasive and metastatic features. However, there is ample evidence to suggest that cycling hypoxia also influences many aspects of tumor progression and therapy resistance [6], [7], [8]. These pioneer works have made oncologists aware of the potential role of cycling hypoxia in tumor progression and treatment.

Cycling hypoxia modulates tumor growth, angiogenic processes, metastasis, and radioresistance in several tumor models [6], [7], [8]. However, some phenotypes seem to be dependent upon tumor type. Different tumor types have distinct effects on cycling hypoxia-mediated tumor progression. To our knowledge, the impact of cycling hypoxia on tumor progression in glioblastoma multiforme (GBM) has not been investigated. Although the detailed mechanism is still undefined, earlier studies have suggested that reactive oxygen species (ROS) and hypoxia-inducible transcription factor 1α (HIF-1α) are potential mediators of cycling hypoxia-mediated tumor progression and radiotherapy resistance [4], [5]. ROS and HIF-1α were shown to be the key mediators of tumor angiogenesis, invasion, and metastasis [9], [10]. However, the lack of direct evidence from the *in vivo* tumor microenvironment is a significant impediment to supporting this notion.

ROS are produced during cycling hypoxia and leads to tumor progression, but the mechanisms of ROS generation and the targets of ROS signals are not well understood. Nox-family NADPH oxidases have proven to be a major source of ROS production in various cell types and have crucial roles in various physiological and pathological processes [11]. Recent studies have demonstrated that NADPH oxidase subunit 4 (Nox4) is expressed in several tumor types such as hepatoma [12], breast cancer [13], ovarian cancer [14], melanoma [15], prostate cancer [16], and various neuroepithelial neoplasms [17], and is involved in cellular senescence, resistance to apoptosis, tumorigenic transformation, cell proliferation, cell survival, and radiation resistance. Strong evidence suggests that these processes are upregulated via Nox4 generation of ROS. Nox4 can also serve as an oxygen sensor to regulate TASK-1 enzyme activity [18] and HIF activity [19]. Based on these data, we hypothesized that Nox4 might be a critical mediator of cycling hypoxia-mediated ROS generation and tumor progression in GBM.

The purpose of this study is to explore the impact of cycling hypoxia on GBM progression and to investigate the potential mechanism of this process using molecular biology and imaging techniques. We have now shows that cycling hypoxic stress significantly increases ROS production, HIF-1 activation, and tumor growth *in vitro* and *in vivo*. Our results also indicate that Nox4 is a critical mediator of these processes and its expression tends to occur in potential cycling hypoxic areas with HIF-1 activation and blood perfusion within the endogenous tumor microenvironment.

## Materials and Methods

### Cell culture

GBM8401 and U87 were cultured in DMEM (Life Technologies) supplemented with 10% fetal bovine serum (FBS), 10 mM HEPES, and 1% penicillin-streptomycin.

### *In vitro* hypoxic treatments

The cells were treated in a Biospherix C-Chamber (Biospherix) inside a standard culture chamber by means of exhausting and gassing with 95% N_2_ and 5% CO_2_ to produce oxygen concentrations of 0.5 to 1% for 4 h at 37°C to achieve non-interrupted hypoxic conditions. For the cycling hypoxic treatment, cell cultures were exposed to 12 cycles of 0.5 to 1% O_2_ for 10 min interrupted by 5% CO_2_ and air for 10 min at 37°C in a hypoxia chamber with a timer-controlled regulator. *In vitro* medium oxygen during cycling hypoxia was determined using the Oxford Oxylite fiberoptic probe (Oxford) and this condition resulted in the medium pO_2_ of 0.8–1.5 mmHg during hypoxic phase

### *In vitro* ROS production

ROS production was assessed by using 10-acetyl-3,7-dihydroxyphenoxazine (Amplex Red, Molecular Probes) to evaluate H_2_O_2_ or carboxy-2′7′-dihydrodichlorofluorescein diacetate (H2DCFDA, Molecular Probes) to assess total ROS. Cells were incubated in phenol-free medium in the presence of 50 µmol/L Amplex Red and 0.1 U/mL horseradish peroxidase or 10 µM H2DCFDA under *in vitro* hypoxic treatments. Fluorescence was measured in a SpectraMax M2/M2e Microplate Reader (Molecular Devices) with excitation at 530 nm and emission at 590 nm for Amplex Red or excitation at 485 nm and emission at 520 nm for H2DCFDA.

### Real-time quantitative PCR

Q-PCR analysis was performed as described previously [20]. The primers for quantitative analysis of Nox4 and the housekeeping gene 60S acidic ribosomal proteins were: Nox4 (F) 5′-ACAGGGGTCTGCATGGTGGT-3′ and (R) 5′-GCAGCCCTCCTGAAACATGC-3′; and the house keeping gene 60S acidic ribosomal protein (F) 5′-ACGAGGTGTGCAAGGAGGGC-3′ and (R) 5′-GCAAGTCGTCTCCCATCTGC-3′.

### Western blot analysis

Whole cell and nuclear extracts were prepared as described previously [20]. Nox4 protein in GBM cells was detected in 150 µg of cell extract by using a monoclonal Nox4 antibody (1∶650; Novus). Nuclear extracts were examined for HIF-1 activation using a monoclonal HIF-1α antibody (1∶750; Novus). Western blots were normalized using a monoclonal anti-β-actin antibody for cell extracts (diluted 1∶10,000; Santa Cruz Technology) and a monoclonal anti-TATA binding protein (TBP) (diluted 1∶2,000; Abcam) for nuclear extracts.

### Small interfering RNA transfection

GBM8401 or U87 cells were transfected with Nox4 small interfering RNA (siRNA) (Santa Cruz Biotechnology) using the OligofectAMINE transfection reagent (Invitrogen) according to the manufacturer's instructions.

### Vector constructions and viral transduction

The lentiviral vector pLKO AS2 (National RNAi Core Facility, Taiwan) served as the backbone to generate a lentiviral vector bearing a luciferase (*Luc*) reporter gene. The *Luc* gene was PCR-amplified from pTA-Luc (Clontech) and inserted into pLKO AS2 under the cytomegalovirus (CMV) promoter at the *Nhe*I and *EcoR*I restriction sites. We used PCR cloning to insert the NESTKGFP∶dMODC [20] fusion reporter gene in place of the original TKGFP fusion reporter gene in dxHRE-tk/eGFP-cmvRed2XPRT [21]. The lentiviral vector pLVCT-tTR-KRAB (Addgene) was used to express Nox4 shRNA (Sigma) following the manufacturer's protocol. Lentivirus or retrovirus production and cell transduction were carried out according to protocols described elsewhere [21], [22]. The GBM8401 and U87 cells bearing the Luc reporter gene and the dual reporter gene cassette were termed GBM8401-Luc or U87-Luc and GBM8401/hif-1-r or U87/hif-1.

### Animal models

Eight-week-old female athymic nu/nu mice were used to establish animal tumor models. For the subcutaneous GBM xenograft model, 5×10^6^ GBM8401-Luc or GBM8401/hif-1-r cells with or without Nox4 short-hairpin RNA (shRNA) transduction were injected subcutaneously into the dorsal aspects of the left anterior limbs and small (80±16.0 mm^3^) subcutaneous tumors developed 14 days later were used for animal imaging studies. For the orthotopic GBM xenograft model, the procedure was carried out following published methods [23]. Briefly, 2×10^5^ GBM8401-Luc cells or GBM8401/hif-1-r cells were harvested by trypsinization and injected into the right basal ganglia of anesthetized mice. Mice bearing the orthotopic GBM8401-Luc or GBM8401/hif-1-r xenograft after 12 days were used for *in vivo* cycling hypoxic stress studies. All animal studies were conducted according to the Institutional Guidelines of China Medical University and approved by the Institutional Animal Care and Use Committees of China Medical University (approval number 97-65-N).

### Animal treatments

Mice bearing the orthotopic GBM8401 reporter xenograft received drinking water containing 5% sucrose only (control), 5% sucrose plus 300 µg/mL Tempol (Tempol treatment), or 5% sucrose plus 2 mg/mL Dox (knockdown) and 24 d of *in vivo* cycling hypoxia. Tumor progression was monitored by weekly bioluminescence imaging and mice were monitored daily for survival. The procedure for *in vivo* cycling hypoxic treatment was carried out following published methods [7], [8]. Briefly, the tumor-bearing mice were exposed to continuous flow of a humidified gas mixture to induce *in vivo* hypoxia in 6-liter hypoxia chambers. The mice were exposed to normal air (control) or 7% O_2_ for 4 h for non-interrupted hypoxic treatment or 12 cycles of 10 min 7% O_2_ breathing interrupted by 10 min periods of normal air breathing for cycling hypoxic treatment. Tumor oxygen was determined with an Oxford Oxylite fiberoptic probe (Oxford) and this condition resulted in the tumor pO_2_ of 1.2–2.5 mm Hg during hypoxic phase. At least 6 mice were used for each cohort. Animals exhibiting significant neurologic compromise, such as limping, or any significant paresis that impaired the ability to obtain food, were euthanized with carbon dioxide gas.

### Animal imaging

The luminescent probe L-012 (Wako Chemical) was administered intravenously (40 mg/kg) after *in vivo* hypoxic treatments for *in vivo* ROS analysis [24]. At 5 min after probe administration, luminescence from the animals was recorded with the IVIS Imaging System 200 Series (Caliper Life Sciences). To image HIF-1 activity, mice were injected with 9.25×10^6^ Bq ^18^F-9-(4-fluoro-3-hydroxymethyl- butyl) guanine (FHBG) and imaged on a small-animal PET scanner (microPET; Concorde Microsystems) [20]. *In vivo* GFP and DsRed expression were measured in an IVIS imaging system 200 series with excitation at 445–490 nm and emission at 515–575 nm for GFP or excitation at 500–550 nm and emission at 575–650 nm for DsRed. The image capture condition was set up as binning (8×8), f2, FOV13, 3 s. Signal intensity after background subtraction was quantified by Living Imaging software. For *in vivo* bioluminescence imaging (BLI) of tumor progression, mice were anesthetized with isoflurane and imaged 15 min after intraperitoneal injection of luciferin. Signal intensity was quantified within a region of interest over the head that was defined with LivingImage software.

### Immunofluorescence imaging

A perfusion marker, Hoechst 33342 (1 mg/mouse; Sigma), was intravenously (i.v.) administered 30 min prior to tumor excision. Tumor tissues were frozen in the OCT embedding matrix (Shandon Lipshaw). Frozen tissue sections (10 µm) were obtained with an OTF cryomicrotome (Bright-Hacker), fixed in ice-cold methanol for 10 min, and washed with PBS. Tumor sections were co-stained for Nox4 by including Nox4 antibody (Novus) at a final concentration of 10 µg/mL. Sections were washed 3 times in PBS, each wash lasting 5 min. For Nox4 staining, sections were incubated with DyLght 649-conjugated goat anti-rabbit antibody (1∶100; Molecular Probes) and washed again. Tissue fluorescence was visualized with the Axio Observer A1 digital fluorescence microscope system (ZEISS).

### Flow cytometry

Tumor tissues were disaggregated with an enzyme cocktail containing collagenase type III (Sigma), hyaluronidase (Sigma), and collagenase type IV (Sigma), washed several times, and resuspended in phosphate-buffered saline (PBS) to produce a single cell suspension. Prior to flow cytometry, cells were incubated with rabbit polyclonal anti-Nox4 antibody in cold fluorescence-activated cell sorting (FACS) buffer (PBS, 0.5% BSA) on ice for 30 min. After washing in FACS buffer, cells were incubated with DyLght 649-conjugated goat anti-rabbit antibody. After the final washing step, fluorescence was measured using a FACScalibur instrument and FACSDiva 6.0 software (BD Bioscience). Tumor cells were gated according to DsRed expression and side scatter (SSC). Nox4 expression was further evaluated after Hoechst 3342 and GFP gating on cycling hypoxic tumor cells (DsRed^+^, Hoechst 3342^+^, and GFP^+^), chronic hypoxic tumor cells (DsRed^+^, Hoechst 3342^−^ and GFP^+^), or normoxic tumor cells (DsRed^+^, Hoechst 3342^+^, and GFP^−^).

### Immunohistochemistry

Tumor tissues were fixed in 4% paraformaldehyde and embedded in OCT compound. Five micrometer sections were immunostained with mouse monoclonal anti-Nox4 (Novus Biologicals), visualized with an AEC kit (InnoGenex), and counterstained with hematoxylin.

### Statistical analysis

For multiple comparisons of nonparametric variables, Kruskal-Wallis ANOVA was used. For parametric variables, ANOVA was used along with Fisher's least-significant-difference (LSD). For survival analysis, statistical software for Kaplan-Meier Survival Analysis with Tarone-Ware statistics (SPSS Inc) was used. *P*<0.05 was considered significant. All analyses were two-tailed.

## Results

### Cycling hypoxia triggers ROS production via Nox4 in glioblastoma cells

**Figure 1:**
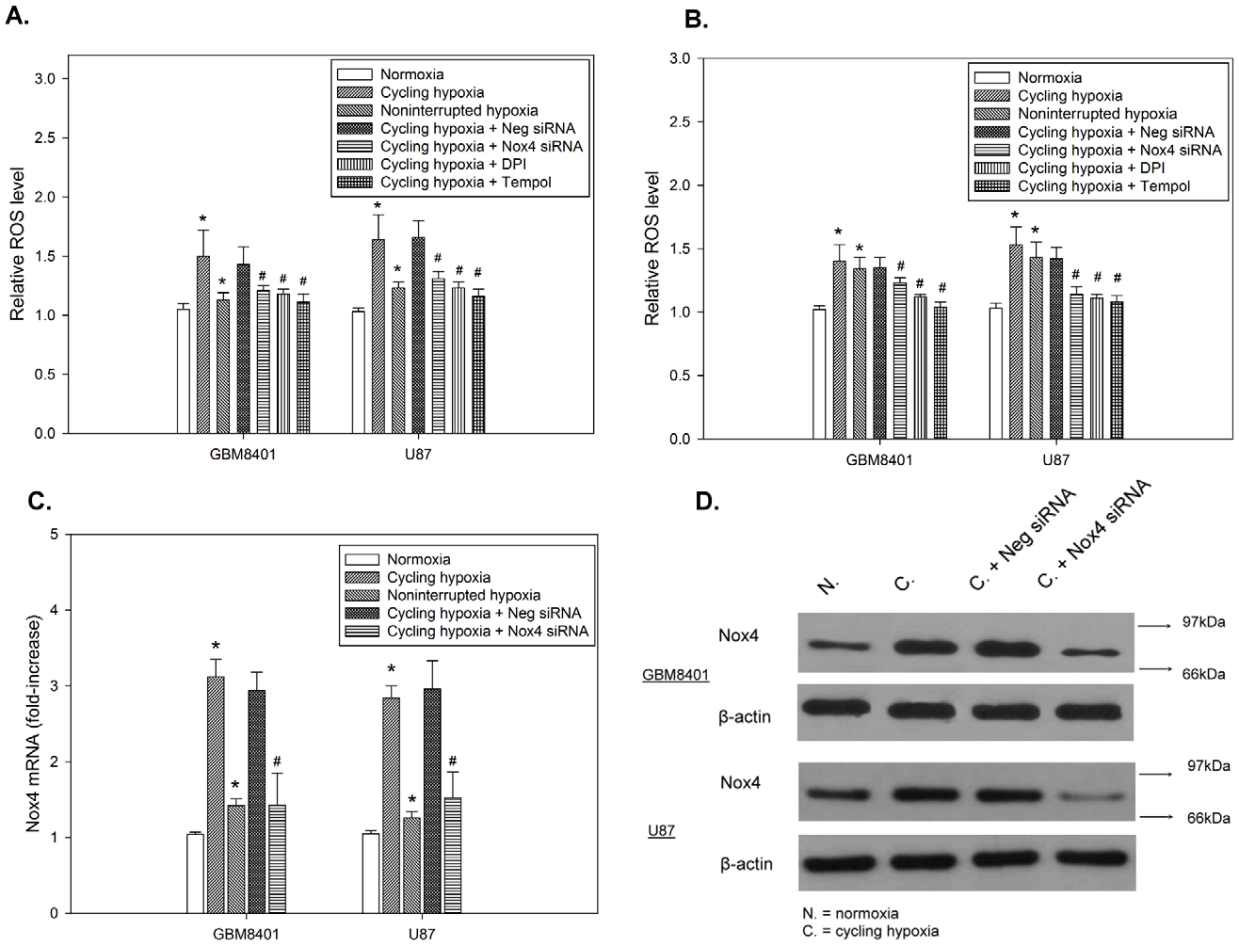
Cycling hypoxia triggers ROS production via Nox4 in glioblastoma cells. GBM8401 and U87 cells were treated with cycling hypoxic stress for 4 h in the absence or presence of Nox4 siRNA or 10 µM diphenyleneiodonium chloride (DPI), and the levels of intracellular ROS (A), H_2_O_2_ (B), Nox4 mRNA (C), and Nox4 protein (D) were evaluated by H2DCHFDA reagent, Amplex Red assay, Q-PCR, and western blotting, respectively. Each bar represents the mean ± standard deviation of triplicate measurements. * p<0.01 compared to normoxia. # p<0.01 compared to cycling hypoxia.

We first examined the effect of experimentally imposed non-interrupted or cycling hypoxic stress on ROS production and Nox4 expression. An increasing fluorescent signal was observed in cycling hypoxia-treated GBM8401 and U87 cells (Fig. 1A and B). In contrast to cycling hypoxia treated-cells, no significant increase in ROS levels was found in normoxic or non-interrupted hypoxic cells. Furthermore, Q-PCR and western blot analysis also showed significantly increased levels of Nox4 mRNA and protein were expressed in cycling hypoxia-treated cells (Fig. 1C and D). To study the source of ROS generation in cycling hypoxia, we used a specific silencing siRNA to knockdown Nox4 induction in GBM8401 and U87 cells under cycling hypoxia. RT-PCR and western blot analysis showed that this siRNA successfully knocked down Nox4 expression, whereas the negative control (Neg) siRNAs did not (Fig. 1C and D). Cycling hypoxia-induced ROS was inhibited by Nox4 knockdown and by treatment with the NADPH oxidase inhibitor diphenyleneiodonium chloride (DPI, 10 µM). These results indicate that cycling hypoxia triggers ROS production via Nox4 in glioblastoma cells.

### Nox 4 knockdown and antioxidant compound suppress cycling hypoxia-induced ROS levels in glioblastoma xenografts

**Figure 2:**
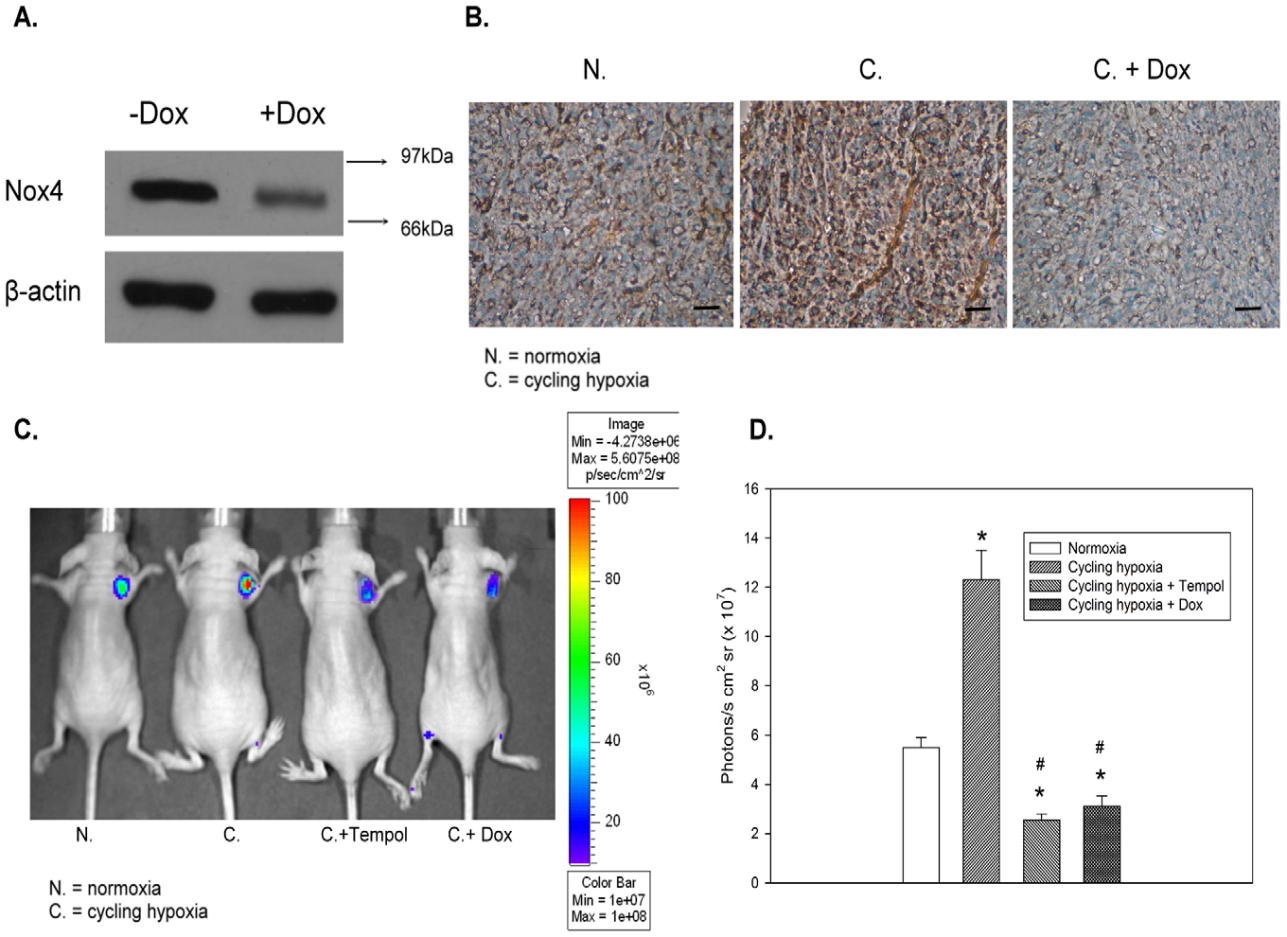
Nox 4 knockdown and a antioxidant compound suppress cycling hypoxia-induced ROS levels in glioblastoma xenografts. (A) Regulation of Nox4 by Dox-inducible shRNA. GBM8401-Luc cells were infected with Tet-regulable lentiviral vectors encoding Nox4 shRNAs. The infected cells were treated with or without Dox for 24 h and harvested for western blot analysis. (B) Immunohistochemical analysis of Nox4 in GBM8401-Luc xenografts with or without conditional knockdown of Nox4 under cycling hypoxic stress. Original magnification, ×200. Bar, 100 µm. (C) *In vivo* optical imaging of GBM-bearing mice injected with L-012. (D) Quantitative data obtained from *in vivo* optical imaging of ROS levels in GBM xenografts with or without Dox or Tempol following *in vivo* cycling hypoxic stress. * p<0.01 compared to normoxia. # p<0.01 compared to cycling hypoxia.

Having linked cycling hypoxia with ROS production, we next sought to determine whether Nox4 knockdown or treatment with an antioxidant compound can suppress cycling hypoxia-induced ROS levels in glioblastoma xenografts. We first utilized tetracycline-inducible lentiviral vectors encoding shRNAs to stably and specifically knockdown Nox4 in GBM8401-Luc cells. These cells expressed a low level of Nox4 in the presence of Dox (Fig. 2A). The Nox4 immunohistochemical analysis in GBM8401-Luc xenografts demonstrated that Dox induction of shRNAs targeting Nox4 *in vivo* also led to suppression of Nox4 expression in glioblastoma tumors (Fig. 2B). As shown in Fig. 2C and D, the ROS levels were significantly higher in cycling hypoxia-pretreated glioblastoma tumors than in control tumors. However, Nox4 knockdown or Tempol treatment in glioblastoma xenografts under cycling hypoxic stress inhibited additional cycling hypoxia and endogenous tumor microenvironment-induced ROS production. These results suggest that cycling hypoxia generates oxidative stress to produce ROS within the tumor microenvironment. Nox4 knockdown or Tempol treatment *in vivo* suppresses cycling hypoxia-induced ROS levels in glioblastoma.

### Cycling hypoxia induces high, long-term HIF-1 activation *in vitro* and *in vivo*

**Figure 3:**
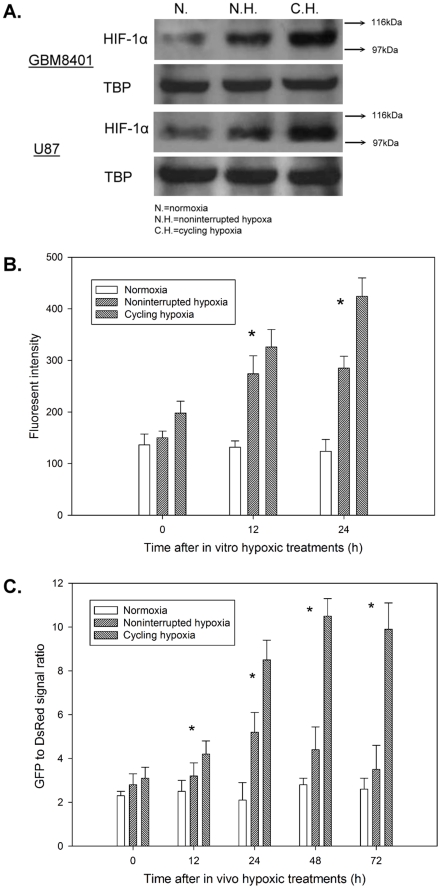
Cycling hypoxia induces higher, long-term HIF-1 activation in glioblastoma cells and xenografts. (A) Western blot analysis of HIF-1α in GBM8401 and U87 cells after cycling hypoxia. Cells were exposed to hypoxic stress, either non-interrupted or cycling, for 4 h and harvested to determine the amounts of HIF-1α protein in nuclear extracts. (B) Transcriptional activity at hypoxia response elements in GBM8401 cells after cycling hypoxic stress. GBM8401/hif-1-r cells were cultured under hypoxic stress, either non-interrupted or cycling, for 4 h and grown in normoxia for different periods, followed by measurements of reporter gene expression. (C) Kinetics of HIF-1 transcriptional activity in GBM8401/hif-1-r xenografts after cycling hypoxic stress. *In vivo* fluorescence imaging (FLI) was performed for GBM8401/hif-1-r tumors before hypoxic treatments and at different times after hypoxic treatments. The data represent the mean ± standard deviation of the ratio of average counts within the tumor region of interest (ROI) in GFP and DsRed signals from 6 mice.

The amount of HIF-1α protein in nuclear extracts was assayed by Western blot analysis after 4 h of *in vitro* hypoxic treatment. Both non-interrupted and cycling hypoxic stress caused GBM8401 and U87 cells to increase expression of HIF-1α protein. However, HIF-1α protein levels in GBM8401 and U87 cells under cycling hypoxic stress were higher than in cells under non-interrupted hypoxic stress (Fig. 3A). We next verified whether this effect could further induce differential HIF-1 signal transduction. As shown in Fig. 3B, transcriptional activity at the hypoxia-responsive elements (HRE) in cycling hypoxia-treated GBM8401/hif-1-r was significantly higher than in the non-interrupted hypoxia-treated group. We then sought to validate our *in vitro* findings in GBM xenografts. *In vivo* optical imaging was used to record reporter activity in 14-day GBM xenografts in mice following *in vivo* hypoxic treatments. Time-course data showed that HIF-1 signal transduction increased steadily over time and peaked at 24 h after non-interrupted hypoxic stress and at 48–72 h after cycling hypoxic stress (Fig. 3C). These data indicate that cycling hypoxic stress results in significantly prolonged elevation of HIF-1 signal transduction in glioblastoma cells.

### ROS is required for cycling hypoxia-induced HIF-1 activation *in vitro* and *in vivo*

**Figure 4:**
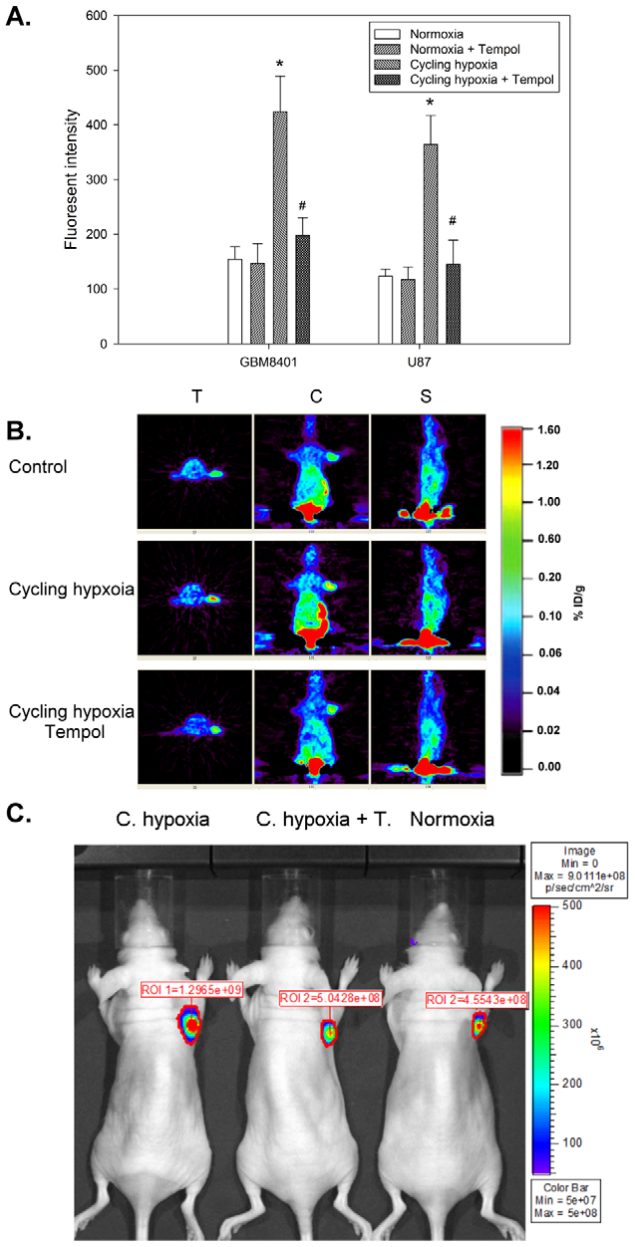
ROS is required for cycling hypoxia-induced HIF-1 activation in glioblastoma cells and xenografts. (A) Flow cytometric analysis of HIF-1 transcriptional activity in GBM8401/hif-1-r and U87/hif-1-r cells exposed to cycling hypoxic stress with or without Tempol. *In vivo* microPET imaging (B) and *in vivo* optical imaging (C) of HIF-1 transcriptional activity in GBM8401/hif-1-r tumors with or without Tempol treatment. MicroPET imaging with [^18^]FHBG and *in vivo* optical imaging were used to determine *in vivo* HIF-1 signal transduction activity 24 h after *in vivo* cycling hypoxia treatment.

To investigate whether ROS is required for cycling hypoxia-induced HIF-1 activation, GBM8401/hif-1-r and U87/hif-1-r cells were treated with Tempol over a 4-h period of cycling hypoxia treatment and Tempol prevented ROS generation in these conditions (Fig. 1A and B). FACS demonstrated that HIF-1 signal transduction activity in the cycling hypoxia-treated cells increased steadily after treatment (Fig. 4A). Tempol treatment following cycling hypoxia abrogated the increase in HIF-1 signal transduction. We then sought to verify our *in vitro* findings *in vivo*. MicroPET and *in vivo* optical imaging studies demonstrated that mice bearing GBM8401/hif-1-r xenografts under cycling hypoxic stress had significantly higher [^18^F]FHBG accumulation and fluorescence intensity in GBM tumors compared to control mice (Fig. 4B and C; Table 1). Moreover, the cycling hypoxia-induced [^18^F]FHBG accumulation and fluorescence intensity in GBM tumors was inhibited by Tempol treatment. These results indicate that ROS are required for cycling hypoxia-induced HIF-1 activation, and Tempol is an effective ROS inhibitor for blocking cycling hypoxia-mediated HIF-1 activation.

**Table 1:** Quantitative data obtained from microPET imaging and *in vivo* optical imaging of HIF-1 transcriptional activity in GBM8401/hif-1-r tumors with or without Tempol treatment.

	MicroPET Imaging (% ID/g)	In Vivo Optical Imaging (×10^9^ p/s/cm^2^/sr)
Normoxia	0.46±0.12	0.43±0.17
Cycling hypoxia	1.32±0.48*	1.13±0.42*
Cycling hypoxia+Tempol	0.57±0.21	0.52±0.18

Each value represents the mean ± standard deviation (SD) of the values obtained from 6 mice.

*p<0.01 compared to normoxia.

### The majority of HIF-1 signal transduction activity and Nox4 expression occurs in endogenous cycling hypoxic areas in solid tumor

**Figure 5:**
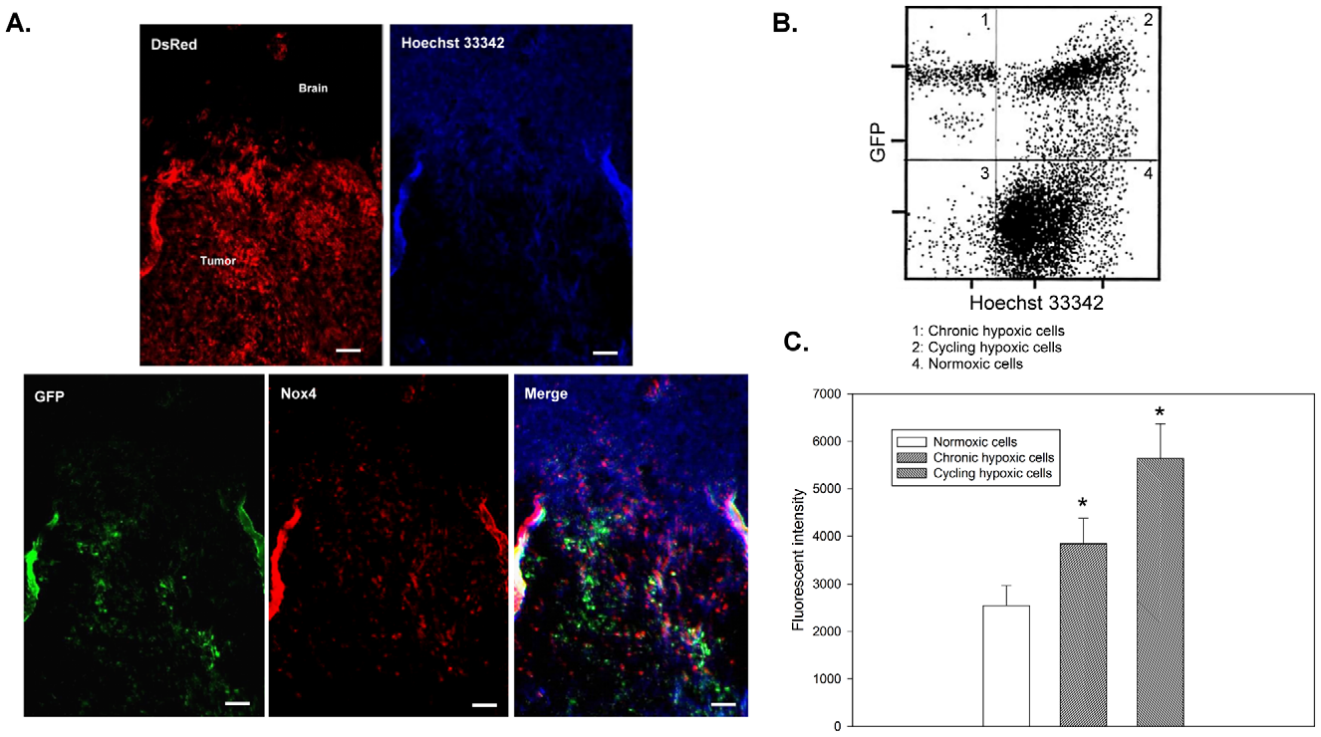
The majority of HIF-1 signal transduction activity and Nox4 expression occurs in endogenous cycling hypoxic areas in a solid tumor. (A) Representative images of microscopic GBM8401/hif-1-r xenografts. Upper left, fluorescence image of DsRed reporter (red), indicating tumor cell localization within the brain. Upper right, fluorescence image of Hoechst 33342 (blue) showing perfusion within the brain and tumor tissue. Lower left, fluorescence image of GFP reporter (green), demonstrating HIF-1 transcriptional activity in tumor cells. Lower middle, fluorescence image of Nox4 staining (red). Lower right, fluorescence overlay image of Hoechst 33342 (blue), GFP reporter (green), and Nox4 (red). Bar, 50 µm. (B) Scatterplots by 2-color staining with Hoechst 3342 and GFP. (C) Mean channel fluorescence of Nox4 staining was determined in cycling hypoxic cells (Hoechst 3342^+^ and GFP^+^), chronic hypoxic cells (Hoechst 3342^−^ and GFP^+^), and normoxic cells (Hoechst 3342^+^ and GFP^−^) as gated in scatterplots by Hoechst 3342 and GFP staining.

To investigate the biosignature of Nox4 expression and HIF-1 signal transduction within the tumor microenvironment, mice bearing 18-d orthotopic GBM8401/hif-1-r xenografts were injected intravenously with a perfusion marker (Hoechst 33342) and the tumors were removed for tissue immunofluorescence imaging. Tight colocalization of higher GFP intensity and Hoechst 33342 signals was observed (Fig. 5A), indicating that the majority of HIF-1 signal transduction occurs in areas with relatively high perfusion. Areas with positive Hoechst 33342 staining and GFP expression were also potential cycling hypoxic areas. However, areas that were positive for GFP expression but negative for Hoechst 33342 were mostly chronic hypoxic areas. Furthermore, Nox4 expression tended to occur in the cycling hypoxic areas but not in the chronic hypoxic areas. To better verify endogenous tumor microenvironment-mediated HIF-1 activation and Nox4 expression in the solid tumor, we identified subpopulations of tumor cells from GBM8401/hif-1-r xenografts based on differential Hoechst 33342 and GFP fluorescence and investigated Nox4 expression in these subpopulations using flow cytometry. As illustrated in Fig. 5B, the tumor suspension consisted of approximately 28±4% cycling hypoxic cells (Hoechst 3342^+^ and GFP^+^), 10±2% chronic hypoxic cells (Hoechst 3342^−^ and GFP^+^), and 58±6% normoxic cells (Hoechst 3342^+^ and GFP^−^). Moreover, Nox4 expression was significantly higher in cycling hypoxic cells than in chronic hypoxic cells or normoxic cells (Fig. 5C). These results suggest that the majority of HIF-1 signal transduction activity and Nox4 expression occurs in areas of endogenous cycling hypoxia in solid tumors.

### Cycling hypoxia promotes tumor growth via Nox4-mediated ROS

**Figure 6:**
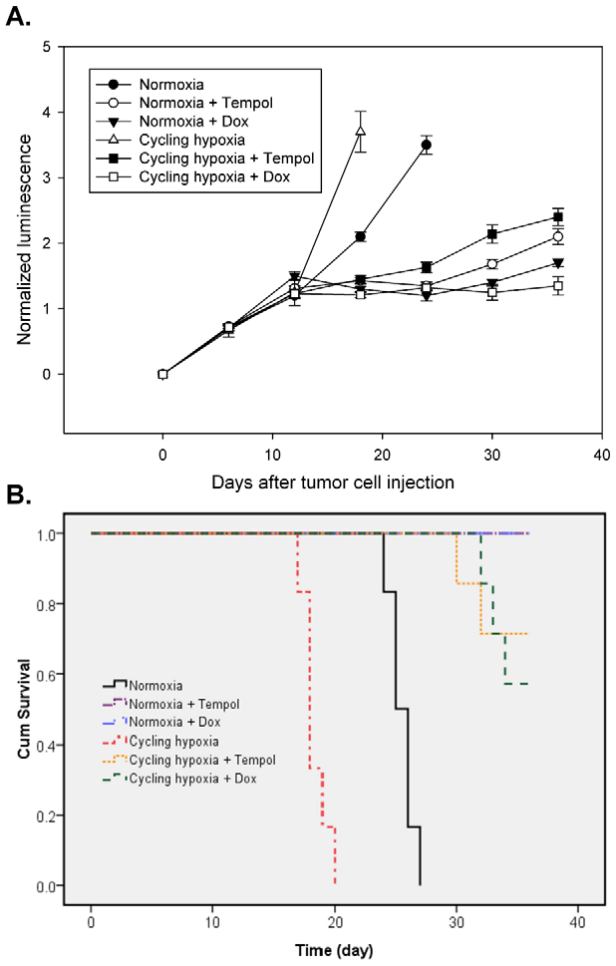
Cycling hypoxia promotes tumor growth via Nox4-mediated ROS in GBM xenografts. (A) The mean normalized BLI values associated with longitudinal monitoring of intracranial tumor growth for each treatment group. Mice bearing 12-d orthotopic GBM8401-Luc xenografts were treated daily with Dox-inducible Nox4 knockdown or 100 mg/kg Tempol following *in vivo* cycling hypoxia treatment for 24 days. Bars report the mean ± standard deviation of measurements in 6 mice. (B) The corresponding survival curves of GBM8401-Luc xenograft-bearing mice exposed to daily treatment with Dox-inducible Nox4 knockdown or Tempol following *in vivo* cycling hypoxia treatment. * p<0.01 compared to normoxia.

We utilized BLI for assessing intracranial tumor response to cycling hypoxia, Nox4 knockdown, and Tempol treatment in the orthotopic GBM8401-Luc xenograft model. There was a highly significant increase in tumor growth rate in the group receiving cycling hypoxia treatment compared to the control group (Fig. 6A). Both cycling hypoxia-pretreated mice and control mice treated with Dox or Tempol showed inhibition of tumor growth. Our results also demonstrated that the mean survival time in cycling hypoxia-pretreated mice was significant lower than in control mice (Fig. 6B). Dox or Tempol treatment significantly prolonged the survival of cycling hypoxia-pretreated mice and normoxic mice. In summary, Nox4 knockdown and Tempol treatment during GBM progression may be a therapeutic approach to block the impact of cycling hypoxia on tumor progression.

## Discussion

Hypoxia and reoxygenation are distinct stimuli capable of stimulating ROS formation. Hypoxia stimulates ROS formation from mitochondria [25] and xanthine oxidase [26], whereas re-oxygenation induces NADPH oxidase-derived ROS formation [27]. However, little is known of the difference in tumor ROS levels between cycling hypoxia and non-interrupted hypoxia and the oxygen sensor that regulates ROS production in the tumor microenvironment has not been identified. Recent studies have demonstrated that Nox proteins are expressed in many cell types and tissues [28]. Their expression and function varies from tissue to tissue. Nox-based oxidases promote cancer development and overproduction of intracellular ROS is thought to increase the risk of cancer [29]. In the present study, *in vitro* and *in vivo* ROS analysis clearly showed that cycling hypoxia could induce more ROS production than normoxia in GBM cells and xenografts. We also examined the role of Nox4 in cycling hypoxia-induced ROS production because it has been suggested that Nox4 is expressed in human glioma [17]. The following evidence demonstrates that Nox4 is essential for cycling hypoxia-induced ROS production. First, cycling hypoxia induced Nox4 mRNA and protein expression in GBM cells. Second, up-regulation of Nox4 expression tended to occur in the cycling hypoxic areas of the solid tumor. Third, knockdown of Nox4 expression or DPI treatment suppressed cycling hypoxia-induced ROS generation. We conclude that Nox4 is a critical mediator of the tumor microenvironment under cycling hypoxia, and mediates ROS production in GBM.

GBM tumors may contain numerous hypoxic areas that exhibit elevated HIF-1 signal transduction activity [30], which results in increased expression of many downstream target genes that contribute to tumor malignancy [31]. Activation of HIF-1 signal transduction in GBM appears to be initiated through a vicious cycle of poorly functioning vasculature that perpetuates the development of chronic or cycling hypoxic regions throughout the tumor [32]. Since it is difficult to explore the naturally occurring variation of different hypoxia-induced responses, we used a direct experimental technique to modify tumor oxygenation and induce additional chronic or cycling hypoxia in tumors. With this approach, we can directly observe the effects of chronic and cycling hypoxia on relevant responses or mechanisms in living subjects. In addition, the dynamics of HIF-1 signal transduction activity mediated by cyclic hypoxia in a tumor is fast due to the instability of the HIF-1α protein under reoxygenation; a reporter gene with a high temporal resolution is required for monitoring such dynamic processes [20]. Although TKGFP has been used for monitoring temporal dynamics and spatial heterogeneity of HIF-1 signal transduction within tumors in living subjects, its use is impractical for real-time monitoring of the dynamics of activity mediated by hypoxia and reoxygenation in tumors because of its poor temporal resolution [21]. To more faithfully reflect the dynamics of HIF-1 signal transduction activity mediated by cyclic hypoxia *in vitro* and *in vivo*, we developed a modified TKGFP (NESTKGFP∶dMODC) for observing the temporal dynamics and spatial heterogeneity of HIF-1 signal transduction activity in tumors. In this study, *in vitro* and *in vivo* data clearly demonstrate that GBM cells or GBM-bearing mice exposed to cycling hypoxia induce more prolonged and higher tumor HIF-1 signal transduction activity than that of non-interrupted hypoxia. Our *in vivo* results validate the *in vitro* results derived from earlier studies [4], [6], [33] and suggest that cycling hypoxia, like chronic hypoxia, can induce HIF-1 transcriptional activity in living subjects.

Although *in vitro* or *in vivo* hypoxic treatments of tumor cells or xenografts can provide indirect evidence of the biosignature of cycling hypoxic cells *in vivo*, it is best to directly validate these biosignatures in the endogenous tumor microenvironment. We have established a reliable protocol of cycling hypoxic cell identification that allows subsequent immunofluorescence imaging or flow cytometric analysis of the biosignature in these cells. We modified a technique based on a previously reported protocol [34], [35]. This technique utilizes the diffusion/consumption properties of Hoechst 3342 when it passes through several cell layers and can separate tumor cells as a function of their distance from the blood supply. However, the original technique cannot be used to distinguish or isolate cycling hypoxic cells and chronic hypoxic cells from a heterogeneous population of tumor cells in the solid tumor due to a lack of a cycling hypoxic biomarker. Here, we identify these cells according to the physiological and molecular characteristics of cycling hypoxia. Cycling hypoxia tended to occur in highly vascular regions with relatively high permeability and therefore, cycling hypoxic areas still have blood perfusion after transient occlusion or narrowing of the vasculature [5], [36]. In contrast, chronic hypoxic areas do not have blood perfusion, even when the blood perfusion of the areas proximal to the blood vessels has been restored. Therefore, the perfusion marker, Hoechst 33342, stains positive in both normoxic and cycling hypoxic cells within solid tumors when the marker is injected into living mice and permitted to circulate for a period of time [34]. Moreover, it has been shown that cells exposed to cycling hypoxia exhibit more robust HIF-1 activation than cells that are chronically hypoxic [6], [33], [37]. Therefore, reporter gene expression is induced by HIF-1 activation in both cycling and chronic hypoxic cells within solid tumors. Cells that are positive for Hoechst 33342 staining and HIF-1 activation are potential cycling hypoxic cells. Therefore, the combination of Hoechst 33342 staining and HIF-1 activation labeling, together with immunofluorescence imaging or flow cytometric analysis, is an effective approach to identifying hypoxic heterogeneous populations in solid tumors.

The effects of chronic hypoxia on HIF-1 regulation have been extensively studied, and it is clear that chronic hypoxia can stabilize HIF-1α due to blockage of the degradation pathway, further inducing its signal transduction activity [38]. Recently, it has been shown that cells exposed to cycling hypoxia exhibit a more robust HIF-1 response than cells that are chronically hypoxic [6]. The results from earlier works, as well as our present results, clearly demonstrate that cells exposed to cycling hypoxia can induce more HIF-1α protein expression and activity than they do under non-interrupted hypoxia. Although the mechanisms of this effect are complex and still not fully clear, ROS may play a role in cycling hypoxia-enhanced HIF-1α protein expression and signal transduction activity [4]. In our current work, *in vitro* and *in vivo* results confirmed that ROS is required for cycling hypoxia-induced HIF-1 activation and the antioxidant compound, Tempol, inhibited cycling hypoxia-induced HIF-1 signal transduction activity. Although the mechanism of ROS-mediated HIF-1 signal transduction activity under cycling hypoxia is still not defined, 2 mechanisms for ROS-mediated HIF-1 activation have been suggested. One possibility is that ROS stabilizes HIF-1α. Early studies demonstrated that the production of ROS under normoxia stabilizes HIF-1α and contributes to HIF-1 activation [39]. The other possibility is that ROS depolymerizes stress granules and further enhances downstream HIF-1 signaling [40]. Pioneer studies showed that a pool of HIF-1-regulated transcripts were kept untranslated in the course of hypoxia in stress granules that were depolymerized during reoxygenation, allowing the rapid translation of sequestered transcripts under normoxia. These mechanisms could also explain, at least in part, how ROS enhances HIF-1 activation and transduction activity under cycling hypoxia.

In earlier studies, cycling hypoxia-promoted tumor invasion was found in animal models [7], [8]. However, the different tumor growth rates between cycling hypoxia-treated mice and control mice were observed in human cervical carcinoma-bearing mice but not in KHT tumor-bearing mice, suggesting that cycling hypoxia has different effects on progression of different tumors. To our knowledge, the impact of cycling hypoxia on tumor progression in GBM has not been investigated. In this study, we found that cycling hypoxia promoted tumor growth in GBM. Importantly, we have also demonstrated that Nox4 and ROS are crucial mediators in cycling hypoxia-promoted tumor growth. Nox4 knockdown or Tempol treatment suppressed tumor ROS and tumor growth in cycling hypoxia-treated mice and control mice. Recently, it has been shown that endogenous ROS play an important role in angiogenesis and tumor growth [10]. Many cancer cells show increased levels of ROS via genetic alternations or growth factors. The increased ROS could modulate signaling pathways and transcription factors for tumor initiation and progression. Here, we highlighted how the tumor microenvironment, cycling hypoxia, increased tumor cell ROS via Nox4 and further promoted tumor growth in GBM. Blockage of ROS production via Nox4 shRNA or Tempol treatment inhibits endogenous tumor microenvironment or exogenous cycling hypoxia-mediated tumor growth, suggesting that ROS play crucial roles in the promotion of tumor growth induced by cycling hypoxia. Although it is possible that other ROS-mediated signaling pathways are involved, we report here that ROS play important roles in cycling hypoxia-mediated HIF-1activation and further promote tumor progression in GBM. This information may be useful to understanding new mechanisms of tumor microenvironment-promoted tumorigenesis and to develop new therapeutic strategies by targeting ROS signaling in human GBM.

### Conclusion

Cycling hypoxia-induced ROS via Nox4 is a critical aspect of cancer biology to consider for therapeutic targeting of HIF-1 activation and cancer progression in GBM.
